# Decoding Brain Development and Function Through GABAergic Inhibitory Neurons

**DOI:** 10.1111/ejn.70650

**Published:** 2026-08-12

**Authors:** Renata Batista‐Brito, Christian Mayer, Mercedes Paredes, Carla G. Silva

**Affiliations:** ^1^ Department of Neuroscience, Friedman Brain Institute Ichan School of Medicine at Mount Sinai New York New York USA; ^2^ Max‐Planck‐Institute for Biological Inteligence Martinsried Germany; ^3^ UCF Neurology Department Neurosciences and Developmental Stem Cell Biology Graduate Programs, and Bi[o]hub San Francisco California USA; ^4^ Translational Neuroscience Department, Brain Division University Medical Centre Utrecht Utrecht the Netherlands

**Keywords:** development, evolution, interneurons, migration, specification, transcription factors

## Abstract

Cortical interneurons are essential for the formation, maturation and functional balance of mammalian brain circuits. Originating in the ventral telencephalon, they migrate into the cortex through organized streams and diversify into multiple inhibitory subtypes. Advances in molecular biology, imaging and single‐cell transcriptomics have revealed the remarkable heterogeneity in their development. Understanding their molecular and functional complexity is a gateway to understanding in full their critical roles in neural synchronization, cognitive processes, sleep regulation and their potential links to the emergence of human‐specific brain functions. These findings aim to inspire future studies focused on deciphering the origins of human interneuron, the diversity of interneuron functions and their implications for understanding neurodevelopmental disorders.

## Graphical abstract

Interneuron subtype specification occurs case by case. Whereas some interneurons are specified very early, at the progenitor level, other interneurons develop final features during migration into the migratory stream or upon integration into specific cortical layers. The concept that interneuron specification can be significantly influenced by external local cues is influencing new studies that are now dedicated to exploring interneuron diversity in the human context and in disease. Human brain development occurs during a protracted period, and interneurons are exposed to a larger number of external cues. In developmental disorders such as autism spectrum disorders and schizophrenia, interneuron specification may be influenced by abnormal developmental programmes. This may permanently change the way interneurons are wired and function.

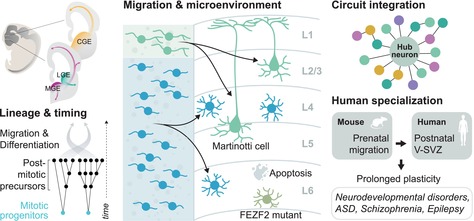

**Abbreviations**
ASD: autism spectrum disorders
CGE: caudal ganglionic eminences
Chodl: chondrolectin
Cxcl12: stromal derived factor 1
CXCR4: C‐X‐C motif chemokine receptor 4
GAD67: glutamate decarboxylase 1
GEs: ganglionic eminences
LGE: lateral ganglionic eminences
MGE: medial ganglionic eminences
nNOS: neuronal nitric oxide synthase
NREM: non‐rapid eye movement
P: postnatal
VIP: vasointestinal peptide
V‐SVZ: ventricular–subventricular zone

## 1 Introduction

The generation and maturation of cortical interneurons have become a central topic in developmental neuroscience over the past several decades, reflecting their critical role in establishing and maintaining balanced neural circuits in the mammalian brain. Early studies revealed that most inhibitory interneurons of the cortex do not originate within the cortex itself but from progenitor regions in the ventral telencephalon, before migrating into the developing cortex. A major conceptual advance was the discovery that these cells travel long distances through organized migratory streams, integrating into the cortex only after extensive tangential migration. Subsequent work demonstrated that cortical interneuron progenitors give rise to diverse interneuron subtypes that contribute to functional brain circuits. Over time, research has increasingly focused on the cellular and molecular mechanisms that guide interneuron migration, positioning, and maturation. More recently, modern approaches such as single‐cell RNA sequencing and in vivo imaging have further refined our understanding of interneuron diversity and developmental trajectories, highlighting the remarkable heterogeneity of inhibitory neurons and the intricate processes that underlie the assembly of cortical circuits. Altogether, inhibitory neurons are a vast population of cells with multiple functions during brain maturation and cognition, such as the onset and regulation of immature patterns of electrical activity, shaping mature electrical activity and synchronization of excitatory neuron assemblies, and even sleep regulation. The field is also beginning to understand how evolutionary specializations and innovations shaped the developmental trajectories of GABAergic interneurons in humans and how they link to human cognition and developmental disorders. Here, we summarize the main findings in the field, including our own studies, and we comment on how further refinement of recent conceptual findings will contribute to understanding how the mammalian brain matures and functions.

## 2 Early Fate Decisions in the Ganglionic Eminences, Establishing the Initial Landscape of Inhibitory Neuron Diversity

How this remarkable diversity is established begins with the earliest fate decisions in the embryonic subpallium. Its foundations are laid in the subpallial progenitor domains, most prominently the medial, caudal and lateral ganglionic eminences (GEs), with additional contributions from adjacent regions such as the preoptic area and septum (Hu et al. 2017; Wonders and Anderson 2006). These territories are patterned by gradients of developmental signalling molecules that establish region‐specific transcription factor programmes, including *Nkx2.1* in the MGE, and *Meis2* in the LGE and Nr2f2 (COUP‐TFII) in the CGE. Progenitors within these patterned domains give rise to largely non‐overlapping classes of GABAergic interneurons and GABAergic projection neurons, thereby establishing a first axis of diversity along spatial coordinates (Lim, Mi, et al. 2018).

Clonally resolved lineage‐tracing studies indicate that individual progenitors can give rise to a spectrum of inhibitory cell types and that this output spectrum shifts according to the progenitor's spatial position within the GEs (Bandler and Mayer 2023). At the molecular level, combinatorial and context‐dependent binding of transcription factors and cofactors at *cis*‐regulatory elements modulates enhancer and promoter activity, shaping the gene expression programmes that drive fate progression and guiding how cells transition through intermediate transcriptional states (Catta‐Preta et al. 2025; Dvoretskova et al. 2024).

Developmental timing constitutes a second axis shaping inhibitory neuron development. Birthdating experiments demonstrated that the embryonic stage at which inhibitory neurons exit the cell cycle influences their eventual subtype identity and laminar allocation (Butt et al. 2005; Miyoshi et al. 2010). More recent findings refine this view by suggesting that birthdate does not only dictate differentiation outcomes but also affects the kinetics of maturation (Bright et al. 2025). Neurons born at different developmental stages traverse transitory cell states at different speeds, effectively adjusting their developmental tempo. Such temporal scaling could allow later‐born neurons to accelerate maturation and align functionally with earlier‐born cohorts, ensuring coordinated integration into emerging circuits despite staggered production.

**FIGURE 1:**
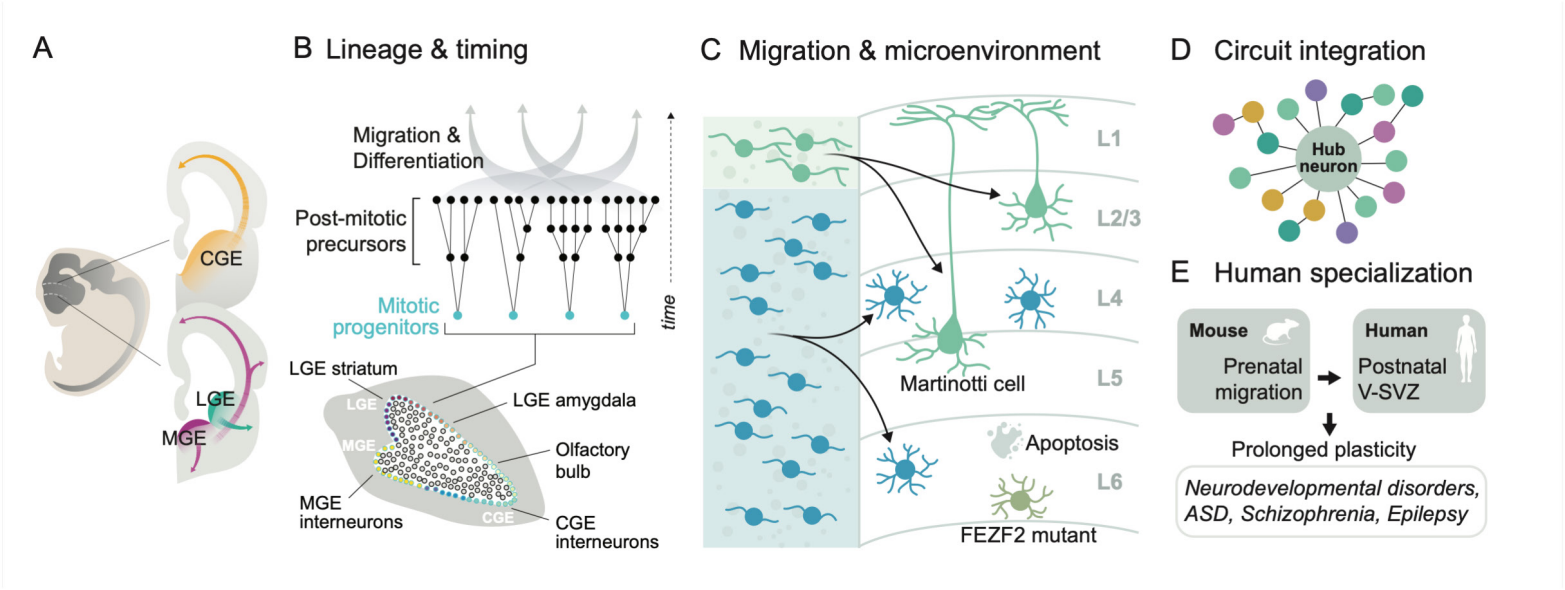
Cortical interneurons are generated in the ganglionic eminences and undergo distinct trajectories starting at the progenitor level (A). The migratory period can condition the fate specification of interneurons as soon as they take decisions on the migratory stream, such as Martinotti cells, or during cortical integration influenced by local cues imposed by projection neurons, as revealed by analysing parvalbumin interneurons in the cortex of *Fezf2* mutants (B). Some interneurons play an important role in shaping cortical networks, acting as hub neurons, recruiting a multitude of other neurons to integrate cortical networks (C). Interneurons in the human brain undergo prolonged plasticity, which may account for vulnerability in neurodevelopmental disorders such as schizophrenia and epilepsy (D).

Spatial identity within the GEs and developmental timing thus define two principal axes of diversity, whereas lineage relationships and combinatorial gene regulation provide the mechanisms operating along them, establishing an initial landscape of biased possibilities. Subsequent processes, including migration, microenvironmental signalling and circuit activity, then act upon this early scaffold to further refine inhibitory neuron identity, function and vulnerability (Figure 1).

## 3 Interneuron Regulation by Microenvironments During Migration and Settling and Its Contribution to Amplifying Interneuron Diversity

Following their generation in the GEs, cortical and hippocampal interneurons travel long distances to reach territories where circuit integration takes place. The molecular regulation of interneuron migration has been extensively studied during the last two decades (Peyre et al. 2015). We know the identity of the major molecular cues for cortical interneurons, acting upon organizing their migration into migratory streams, the molecular roads linking the GEs to the forebrain. Lim and colleagues found the first compelling evidence that interneuron subtype specification may occur as early as some progenitors are specified or as soon as the choice of the migratory stream is made during early embryogenesis (Lim, Pakan, et al. 2018; Mayer et al. 2018).

After the period of migration, interneuron molecular diversification is largely amplified. Variable cues acting on immature interneurons during variable temporal windows bring numerous possibilities to influence interneuron outcomes. Although there is evidence that interneuron subtype specification may depend on extracellular cues, how microenvironments in the maturing cortex and hippocampus mechanistically contribute to this process remains more enigmatic (Lodato et al. 2011). The erratic pattern of interneuron migration during the last phase of radial migration, around birth and the early postnatal period, may facilitate the random exploration of diverse cellular and molecular environments (Guo and Anton 2014). Transplantation studies revealed that different interneuron types can be permissive to the extracellular environment to different extents (Quattrocolo et al. 2017). In a recent elegant study using the *Fezf2* mutant in which there is a loss of Layer 5 and a gain of Layer 6 projection neurons, it was shown that the proportion of deep‐layer parvalbumin and somatostatin interneurons changes due to distinct mechanisms (Wu et al. 2026). Whereas somatostatin interneurons die by apoptosis, parvalbumin interneurons are transformed from one subtype to another. The authors hypothesize that such differences may be linked to the maturation profile of somatostatin and parvalbumin interneurons. Because parvalbumin maturation is slower, these interneurons remain more ‘plastic’ and hence more permeable to the extracellular environment. Another interesting finding of the study is that within the same population of somatostatin interneurons that undergo programmed apoptosis, some interneurons survive and adapt, suggesting that this process is not entirely rigid. It will be interesting to explore if the more malleable somatostatin interneurons were generated at a later developmental stage, and hence, this property is related to their maturation state.

Altogether, these studies stress the relevance of analysing interneuron molecular properties and function in a space‐ and time‐dependent manner. The challenge in the next years will be to dissect the instructive cell types, timing, signals and molecular pathways involved in interneuron specification and final feature acquisition. This is not an easy task because extracellular cues may influence interneurons' destination, their survival or the acquisition of specific molecular profiles underlying function. A new direction of the field will be to decipher which interneuron populations are more susceptible to being changed by genetic and environmental factors in neurodevelopmental disorders and how these conditions alter their trajectories and function. It will also be important to explore whether interneuron modifications result from direct signalling from the altered genetic or extracellular microenvironments or instead represent compensatory attempts to restore the excitation/inhibition balance in neuronal circuits.

## 4 Inhibitory Neurons and Internal Dynamics

One fascinating aspect of neuronal circuit formation is the integration of inputs from the outside world onto internally generated dynamics (neuronal activity in the absence of external stimuli or behavioural task demand). Developing brain circuits exhibit prominent spontaneous activity, characterized by recurring synchronous network events that are initially driven by self‐generated peripheral inputs, but progressively decouple from external stimuli and become intrinsically generated (Martini et al. 2021; Molnár et al. 2020). In mice, the second postnatal week (P10–P14) marks a major developmental transition characterized by coordinated changes in neural circuitry and behaviour. During this period, cortical recurrent connectivity strengthens both structurally and functionally (Ibrahim et al. 2021; Ko et al. 2014; Pouchelon et al. 2021), network activity progressively decouples from peripheral drive (Dard et al. 2022; Gribizis et al. 2019), and highly connected ‘hub’ neurons emerge within cortical circuits (Bocchio et al. 2020; Bollmann et al. 2023; Dard et al. 2022; Duan et al. 2020; Modol et al. 2020). In parallel, subcortical neuromodulatory systems begin to regulate brain activity in a state‐dependent manner, coinciding with the onset of slow‐wave (NREM) sleep (Blumberg et al. 2022). During this period, GABAergic population dynamics exhibit a transiently organized structure, forming coordinated cortical interneuron assemblies that dissipate in a sensory‐dependent manner with the onset of active sensation (Modol et al. 2020). This transient patterning is thought to be orchestrated by hub neurons and coincides with a pronounced suppression of bottom‐up sensory input, initially generated in peripheral organs, alongside the emergence of intrinsically driven cortical activity (Baruchin et al. 2023; Cossart and Garel 2022; Dard et al. 2022; Gribizis et al. 2019). We refer to this developmental window (P10–P14) as a phase of ‘developmental internalization’, during which a functional scaffold is established to prepare the cortex for externally driven sensory processing, prior to the onset of active exploration.

Although developmental hub neurons have been identified in both ex vivo and in vivo preparations (Allène et al. 2008; Bocchio et al. 2020; Bollmann et al. 2023; Dard et al. 2022; Duan et al. 2020; Modol et al. 2020; Picardo et al. 2011), their cellular identity remains largely defined at the level of GABAergic neurons. In contrast to most cortical GABAergic inhibitory neurons, which typically project locally within restricted cortical domains (Tremblay et al. 2016), hub neurons exhibit long‐range projections spanning extensive regions of the hippocampus and neocortex (Tomioka et al. 2005). They are highly connected within local circuits, possess widespread axonal arbours and are characterized by the expression of markers such as SST, M2 muscarinic receptors and nNOS (Cossart and Garel 2022; Dittrich et al. 2012; Echagarruga et al. 2020; Endo et al. 2016; Morairty et al. 2013; Perrenoud et al. 2012; Picardo et al. 2011; Williams et al. 2018). Their distinctive connectivity and morphology likely enable them to interact with a disproportionately large number of neurons, thereby exerting a strong influence over cortical network dynamics.

Prior work has shown that in adulthood there is a population of cortical GABAergic cells that express somatostatin, the muscarinic receptor m2, and neuronal nitric oxide synthase and chondrolectin (named somatostatin‐Chodl) that have long‐range axons that simultaneously target multiple cortical regions. Given their long‐range projections and marker expression, we hypothesize that somatostatin‐Chodl cells serve as developmental hub neurons, causing internally generated dynamics during a restricted but critical developmental period marked by the inhibition of self‐generated peripheral inputs. In future work, it will be interesting to test this hypothesis.

During active sleep, neural activity arising from the sensory periphery (e.g.*,* evoked by sleep‐dependent myoclonic twitches or spontaneous retinal waves) is conveyed to the thalamus and cortex, leading to the idea that active sleep represents a training programme for the cerebral cortex, providing bottom‐up information that instructs and informs the developing circuits about the receptive fields of sensory receptor systems (Blumberg et al. 2022). During the second postnatal week, sleep patterns radically change with the emergence of slow‐wave sleep (or NREM sleep) (Rensing et al. 2018). This transition is concomitant with cortical activity transitioning from being driven by the periphery to be internally driven (Cossart and Khazipov 2022; Gribizis et al. 2019), with the emergence of hub neurons (Bocchio et al. 2020; Cossart and Garel 2022; Cossart and Khazipov 2022; Modol et al. 2020; Picardo et al. 2011) and with cortical activity being modulated by the behavioural state of the animal (Colonnese et al. 2017; Cossart and Khazipov 2022; Murata and Colonnese 2018). Interestingly, prior work shows that in adulthood, somatostatin‐Chodl cells are selectively active during low‐arousal states and largely silent during periods of high arousal and that, in contrast to most neocortical inhibitory neurons, and despite their sparsity, exert widespread influence across the neocortex via long‐range axons that simultaneously target multiple regions. Selective activation of somatostatin‐Chodl cells is sufficient to promote multi‐region cortical synchronization characteristic of low‐arousal states and to induce sleep (Ratliff et al. 2024). Here, we propose that somatostatin‐Chodl cells are a key circuit element underlying the emergence of NREM sleep during the second postnatal week. Testing this hypothesis would not only identify a specific cellular substrate for this developmental transition but also demonstrate how a single inhibitory neuron subtype can exert broad influence over brain state and network dynamics.

## 5 Human Interneurons and Disease

Although rodent studies have established foundational principles of interneuron specification and migration (Wonders and Anderson 2006), emerging evidence demonstrates that human interneuron development follows a markedly prolonged and specialized trajectory. Studies of human tissue have revealed substantial differences in developmental timing, migratory dynamics, and subtype composition. These findings challenge traditional views that cortical circuit assembly is predominantly prenatal and suggest that inhibitory network construction in humans extends well into the neonatal period. The infant human brain, for example, retains substantial populations of immature, migratory GABAergic interneurons within an expanded ventricular–subventricular zone (V‐SVZ). Concurrently, the human cortex exhibits expanded subtype diversity, especially among caudal GE‐derived interneurons, that could lead to the expansion of subtype‐specific populations such as vasointestinal peptide (VIP)–expressing cells. These human‐specific features may support prolonged cortical plasticity but also introduce windows of vulnerability linked to neurodevelopmental and neuropsychiatric disorders such as epilepsy, autism spectrum disorder (ASD), schizophrenia and intellectual disability.

The human V‐SVZ exhibits structural features not observed in rodents. Sanai and colleagues described a distinctive, multilayered organization including an expanded outer SVZ (Sanai et al. 2004). Subsequent work demonstrated that late‐gestation and neonatal human brains contain abundant doublecortin‐positive migratory neuroblasts within this region. Referred to as the ‘Arc’, this area contains immature GABAergic interneurons that migrate postnatally into frontal and temporal cortices (Kim et al. 2025; Paredes et al. 2016). Time‐lapse imaging and histological analyses confirm that these neurons exhibit active migratory behaviour after birth, forming organized streams directed towards higher association regions. This prolonged migration contrasts sharply with rodents, where interneuron positioning is largely finalized prenatally. Transcriptomic data further reveal regional heterochrony in cortical maturation. The human temporal lobe shows delayed transcriptional maturation compared to the frontal cortex, with prolonged expression of genes associated with neurogenesis, microtubule organization and neuronal migration (Kang et al. 2011). It is an exciting possibility, yet without experimental validation, that continued interneuron recruitment during infancy may contribute to extended plasticity in regions involved in language, auditory processing and higher‐order cognition.

In rodents, MGE‐derived parvalbumin and somatostatin interneurons constitute the largest group of cortical inhibitory cells. CGE‐derived interneurons—including VIP, calretinin and reelin subtypes—represent a smaller fraction (Wonders and Anderson 2006). In contrast, primate and human studies suggest an increase in the proportion of CGE‐derived populations. Comparative developmental analyses indicate augmented CGE contribution to primate cortex (Hansen et al. 2013; Ma et al. 2013). Single‐cell and single‐nucleus transcriptomic studies of human cortex demonstrate increased transcriptional diversity and subtype heterogeneity compared to mouse (Hodge et al. 2019; Nowakowski et al. 2017). VIP‐expressing interneurons, typically CGE‐derived, have been proposed to be enriched in human association cortices. These cells frequently participate in disinhibitory circuits, targeting other inhibitory interneurons and modulating cortical plasticity. Cortical GABAergic interneurons generated in the ventral developing brain travel long distances to their final destinations. Although there are examples of interneuron migration in the neonatal human brain, the extent of postnatal migration across species and its contribution to cortical interneuron diversity, remains unknown. Transcriptomic and histological approaches revealed that Arc neurons are diverse interneurons from the medial and caudal GEs that migrate into the frontal, cingulate, and temporal cortex. Arc–cortical targets exhibit an increase in VIP neuronal density compared to other regions. Our findings support the Arc supports the expansion of postnatal neuronal migration for cortical interneuron patterning in gyrencephalic brains (Kim et al. 2025). Because CGE‐derived interneurons are generally late‐born and mature more slowly than parvalbumin interneurons, their expansion in humans may create extended windows of circuit refinement. The preferential presence of CGE‐associated transcriptional programmes in neonatal migratory streams suggests that postnatal interneuron recruitment may selectively shape the inhibitory architecture of the human cortex. Such expansion in gyrencephalic brains may represent an evolutionary adaptation supporting flexible cognition and prolonged learning.

Several features distinguish human inhibitory neuron development from rodent models. Firstly, the structural complexity of the human V‐SVZ provides a specialized niche for postnatal neuroblast populations. This supports the interneuron migration that persists into infancy, particularly targeting frontal and temporal regions. Secondly, human interneurons exhibit greater molecular heterogeneity within major subclasses than observed in mouse cortex (Bakken et al. 2021; Hodge et al. 2019; Shi et al. 2021). Finally, functional maturation appears delayed; human interneurons show prolonged intrinsic and synaptic developmental timelines relative to rodents (Molnár et al. 2020). Together, these features suggest that inhibitory circuit assembly in humans is temporally stretched and regionally specialized. Rather being fully established at birth, inhibitory networks continue to be constructed during early life, potentially aligning circuit maturation with environmental input and experience.

Despite recent advances, major gaps remain in understanding human interneuron biology. The molecular mechanisms guiding postnatal migratory streams are incompletely understood. Although embryonic guidance cues such as CXCR4–CXCL12 signalling are well characterized in rodents, their precise roles in prolonged human migration requires clarification. The extent to which vascular signals, extracellular matrix components and activity‐dependent mechanisms regulate late‐stage migration is also unclear. Another unresolved question concerns the long‐term fate of postnatally migrating interneurons. The proportion that survives, integrates functionally and contributes to adult inhibitory networks remains uncertain. Additionally, many rodent‐based paradigms may not fully capture human‐specific subtype expansion and developmental timing. Emerging organoid systems and gyrencephalic animal models offer promising avenues, but direct in vivo human mapping remains limited.

Defects in inhibitory circuit development are strongly implicated in neurodevelopmental and psychiatric disorders. In ASD, single‐cell transcriptomic studies reveal cell‐type–specific alterations in interneuron gene expression (Velmeshev et al. 2019). Imbalance between excitation and inhibition is widely hypothesized to underlie sensory and cognitive phenotypes. In schizophrenia, reduced expression of parvalbumin and GAD67 in cortical interneurons has been consistently observed (Lewis et al. 2005). Such alterations may disrupt gamma oscillations and impair cortical synchronization. Epilepsy similarly involves dysfunction or loss of inhibitory interneurons, leading to hyperexcitability.

The prolonged developmental timeline of human interneurons introduces additional vulnerability. Perinatal hypoxic injury, for example, may disrupt migratory dynamics or subtype maturation during critical windows without producing overt structural lesions. An exciting possibility is that because CGE‐derived interneurons are recruited postnatally, they may contribute to disinhibitory and plasticity‐regulating circuits. It remains to be tested whether disturbances during infancy may be linked to lasting consequences for language, executive function and social cognition. A hypothesis is that the same extended developmental window that enables flexible circuit refinement may increase susceptibility to environmental and genetic perturbations. Future research integrating human transcriptomics, advanced imaging and clinically informed models will be essential for understanding how inhibitory circuit assembly shapes cognition and disease. The uniquely prolonged and diversified development of human interneurons reframes early life as an active and dynamic period of inhibitory network construction, rather than a phase of mere refinement.

## 6 Conclusion

‘The mysterious butterflies of the soul’, first recognized by Ramόn y Cajal by their visual complexity, are now understood to describe inhibitory neurons. The specialization of imaging and sequencing tools have corroborated the truths of his fascination with these cells. Yet even he would not have predicted the diverse and intricate roles of this special group of neurons. Inhibitory neurons are born in diverse neurogenic niches in the brain and, instead of being limited to their site of origins, contribute to the circuitry across different areas of the brain. Functional studies have shown that this distribution is necessary for neurotypical cognitive functions and behaviours, including sleep. Given their importance, inhibitory neurons need to be studied at multiple levels: their developmental regulation, cell fate restrictions and the breadth of their function.

## Author Contributions

**Renata Batista‐Brito:** conceptualization, writing – original draft, validation, visualization, writing – review and editing, supervision. **Christian Mayer:** conceptualization, writing – original draft, validation, visualization, writing – review and editing, supervision. **Carla G. Silva:** conceptualization, writing – original draft, validation, visualization, writing – review and editing, supervision. **Mercedes Paredes:** conceptualization, funding acquisition, validation, visualization, writing – review and editing, supervision.

## Funding

The authors have nothing to report.

## Ethics Statement

Ethical approval was not required for this study because it is a review article based exclusively on previously published literature.

## Conflicts of Interest

The authors declare no conflicts of interest.

## Data Availability

This article is a review of previously published studies, and no new datasets were generated or analysed during the current study.
